# Neurological imaging findings in hospitalized COVID-19 patients: a retrospective observational study in two Brazilian reference centers

**DOI:** 10.1590/0004-282X-ANP-2021-0103

**Published:** 2022-02-21

**Authors:** Angelo Chelotti DUARTE, Ricardo Hiroshi Murashita FUJIKI, Larissa Freitas Peixoto GLÓRIA, Diego Cardoso FRAGOSO, Felipe Torres PACHECO, Camila CALVI, Marcos ROSA-JÚNIOR, Antonio Carlos Martins MAIA, Antônio José da ROCHA

**Affiliations:** 1 Irmandade da Santa Casa de Misericórdia de São Paulo, Departamento de Radiologia, São Paulo SP, Brazil.; 2 Hospital Estadual Jayme Santos Neves, Departamento de Radiologia, Serra ES, Brazil.; 3 Fleury Medicina e Saúde, Departamento de Neurorradiologia, São Paulo SP, Brazil.; 4 Diagnósticos da América S.A., Departamento de Imagens Médicas, Divisão de Neurorradiologia, São Paulo SP, Brazil.; 5 Universidade Federal do Espírito Santo, Hospital Universitário Cassiano Antônio Moraes, Divisão de Neurorradiologia, Vitória ES, Brazil.

**Keywords:** COVID-19, Stroke, Neurology, COVID-19, Acidente Vascular Cerebral, Neurologia

## Abstract

**Background:**

A variety of neurological manifestations have been attributed to COVID-19.

**Objective:**

To investigate the occurrence of neurological symptoms and neuroimaging findings in patients hospitalized in two Brazilian reference centers.

**Methods:**

We performed a retrospective cohort study of patients who had laboratory-confirmed COVID-19 presenting in two hospitals in Brazil between March 4 and July 7, 2020, who underwent brain imaging.

**Results:**

We recorded 1,359 patients with laboratory-confirmed COVID-19. Brain imaging was performed in 250 (18.4%) patients with neurological symptoms, and nine of them (3.6%) had acute or subacute ischemic stroke neuroimaging findings. Six of the nine patients initially presented with respiratory symptoms while the other three patients presented to the emergency room with acute stroke signs.

**Conclusions:**

We described the neuroimaging findings of patients infected with COVID-19 who presented with neurological symptoms in two major hospitals in Brazil. We reinforce the importance of being aware of cerebrovascular complications, both in severe hospitalized patients and in patients who present to the emergency room with acute neurological symptoms, even in the elderly.

## RESUMO

**Introdução:**

Diversas manifestações neurológicas têm sido atribuídas à COVID-19.

**Objetivo:**

Investigar a ocorrência de sintomas neurológicos e achados de neuroimagem em pacientes internados em dois centros de referência brasileiros.

**Métodos:**

Estudo retrospectivo que avaliou pacientes internados em dois hospitais no Brasil entre 4 de março e 7 de julho de 2020, com confirmação laboratorial de infecção pelo COVID-19 e que foram submetidos a tomografia computadorizada ou ressonância magnética do crânio em razão de sintomas neurológicos.

**Resultados:**

Obtivemos 1.359 pacientes com confirmação laboratorial de infecção pelo COVID-19. Deles, 250 (18,4%) apresentaram sintomas neurológicos e foram submetidos a exames de imagem do crânio e nove (3,6%) demonstraram achados de neuroimagem compatíveis com acidente vascular cerebral (AVC) isquêmico agudo ou subagudo. Em seis dos nove pacientes, observaram-se inicialmente tosse e dispneia graves, enquanto outros três chegaram ao pronto-socorro com sinais de AVC agudo.

**Conclusões:**

Foram descritos os achados de neuroimagem em pacientes infectados com COVID-19 em dois grandes centros de referência brasileiros. Reforçamos a importância de estar atento aos quadros neurológicos, especialmente de AVC, tanto em pacientes graves hospitalizados quanto em pacientes que chegam ao pronto-socorro com sintomas neurológicos agudos, mesmo em idosos.

## INTRODUCTION

In December 2019, an outbreak of coronavirus disease (COVID-19) began in Wuhan, China. Due to its high transmissibility, the disease spread worldwide, and was officially declared a pandemic by the World Health Organization (WHO) on March 11, 2020^1^.

The first case of COVID-19 in Brazil was officially confirmed on February 26, 2020, and the community transmission stage occurred on March 20, 2020^1^. To date, Latin America, and Brazil in particular, has been one of the epicenters of the disease, ranking second only to the United States with the most cases worldwide (about 19,200,000 cases and 536,000 deaths until the second half of July 2021)^2^.

The plethora of manifestations of the SARS-CoV-2 infection is not entirely understood with several clinical manifestations reported in the literature, such as encephalopathy, cerebrovascular disease, epilepsy, neuromuscular disease, anosmia, even Guillain-Barré syndrome but the association between COVID-19 and neurological symptoms has been recognized^3^ ^,^ ^4^ ^,^ ^5^ ^,^ ^6^ ^,^ ^7^. Thus, this study’s objective was to investigate the neurological symptoms and neuroimaging findings in COVID-19 infection.

## METHODS

This retrospective study was conducted in two tertiary-care Brazilian hospitals, one in São Paulo-SP and the other in Serra-ES. The government of Espírito Santo supports the Hospital in Serra, an exclusive referral center for patients with COVID-19 during the pandemic. Inclusion criteria were patients hospitalized for COVID-19 symptoms who developed stroke and patients who had a stroke, and in both cases patients should have a positive reverse transcriptase-polymerase chain reaction (rRT-PCR) for novel coronavirus infection.

Hospitalized patients with confirmed COVID-19 (via rRT-PCR of respiratory secretions) admitted at SCMSP between March 14 and June 3, 2020, and at HEJSN between April 4 and July 7, 2020 were considered. All patients presenting acute neurological symptoms during hospitalization that required brain imaging investigation and positive rRT-PCR were included. Brain imaging with low quality or with artifacts that prevented analysis were excluded.

All exams were performed based on clinical indications. Computed tomography (CT) imaging was performed in a Toshiba Alexion 16-slice scanner (Toshiba Medical Systems, Nasu, Japan) at HEJSN and in a Philips Brilliance 64-slice scanner (Philips Medical, Eindhoven, The Netherlands) at SCMSP. CT protocols of the two institutions were similar, with 0.625-mm section thickness, 22 cm FOV, 120 kV (peak), and 250-300 mA. Coverage was from the foramen magnum to the vertex with posterior multiplanar reconstruction. Magnetic resonance (MR) imaging was performed only at SCMSP with a Philips Achieva (1.5T) scanner. The imaging protocol included diffusion-weighted imaging (DWI), susceptibility-weighted imaging (SWI), volumetric fluid-attenuated inversion recovery (FLAIR), and intracranial time-of-flight (TOF) MR angiography. The research ethics committee approved the study in both institutions.

Neuroimaging was reviewed in consensus by two certified neuroradiologists, one with 20 and the other with 10 years of experience (ACMMJ, FTP). Patients with a stroke diagnosis were classified into three degrees of certainty (possible, probable, and confirmed) of COVID-19-associated stroke according to the revised definition proposed by Vogrig et al.^8^. Clinical information was collected from the electronic medical records (age, sex, initial symptoms, and outcome after two weeks).

## RESULTS

One thousand three hundred and fifty-nine patients with laboratory-confirmed COVID-19 were evaluated. Neuroimaging was required for 250 (18%) patients who presented neurological symptoms; 239 (95%) patients had brain CT, 11 (4%) patients had MR imaging, and seven (3%) patients performed both studies. All clinical manifestations with imaging findings were attributed to cerebrovascular disease. Enhanced CT was performed only in 4 patients, who had positive findings on the non-enhanced CT.

**Figure 1.**
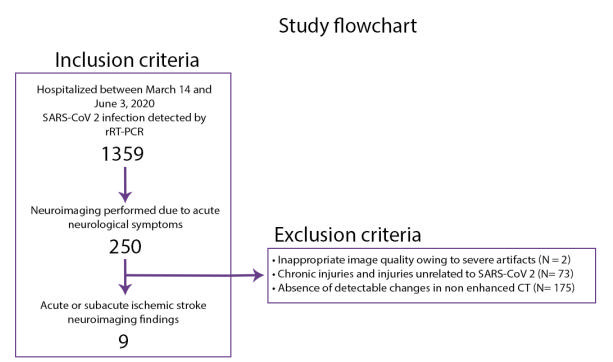
Inclusion and exclusion criteria flowchart.

In our cohort, there was no evidence of other neurological manifestations associated to COVID-19 infection such as encephalitis, Guillain-Barre syndrome, acute disseminated encephalomyelitis, and acute necrotizing encephalopathy^9^ ^,^ ^10^ ^,^ ^11^ ^,^ ^12^. Nine of the 250 patients (3.6%) had acute or subacute ischemic stroke neuroimaging findings (Figure 1), and four performed the enhanced CT. In this patient cohort, the age range was 53-79 years, the median age was 69 years, and 66% were men.

Respiratory symptoms such as severe cough and dyspnea were the main reason for the hospitalization of six patients (6/9). They subsequently developed altered state of consciousness and acute neurological signs. The average time between the onset of respiratory symptoms and the imaging evaluation for the acute neurological sign was 12 days (3-23 days).

**Figure 2.**
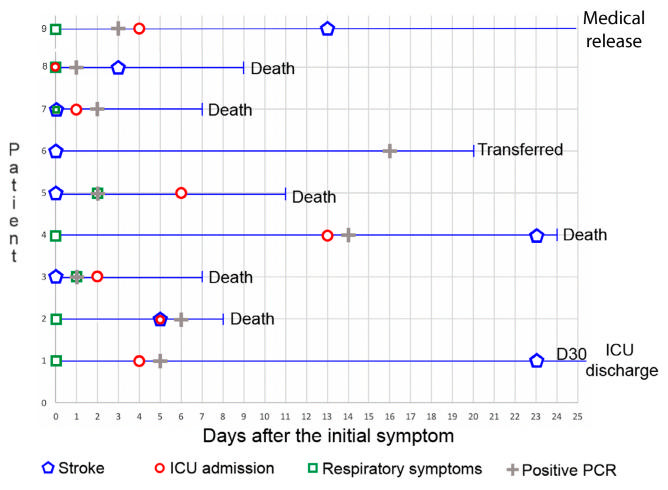
Patients’ symptoms and clinical status timeline.

Acute neurological signs were the main reason for hospitalization of the remaining patients (3/9) who arrived without respiratory symptoms. The three patients had hemiparesis; one patient had syncope and one also had dysarthria and altered state of consciousness. One of these patients developed cough and dyspnea after two days of hospitalization, while the other two remained asymptomatic from the respiratory point of view. The detailed timeline is represented in Figure 2.

ICU: intensive care unit.

**Figure 3.**
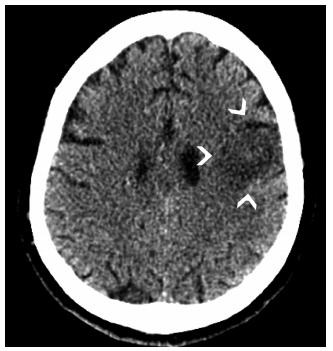
Patient 6. Axial unenhanced computed tomography - ischemic stroke acute findings compromising cortical and subcortical white matter of the left middle frontal gyrus (arrowheads).

**Figure 4.**
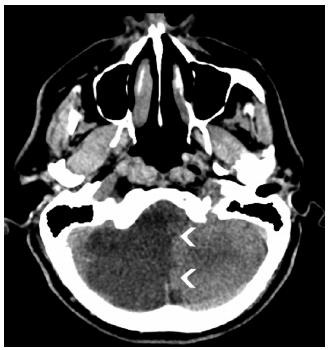
Patient 5. Axial unenhanced computed tomography - hypoattenuating area involving the posteroinferior right cerebellum (arrowheads), related to ischemic stroke involving posteroinferior cerebellar artery.

All neurovascular imaging findings (Figures 3 and 4) were related to ischemic stroke. Six patients (66%) had posterior territory infarction (three in the posterior inferior cerebellar artery territory, two in the basilar artery territory, and one in the posterior cerebral artery territory). Two patients (22%) had an anterior cerebral artery infarction and one had a middle cerebral artery. Almost all patients (89%) had comorbidities related to an increased risk of stroke (Table 1).

**Table 1.** SARS-CoV-2 associated stroke - location and clinical information.

Patient	Age and sex	Stroke location	Schematic Figure	Associated findings	Comorbidities	D-dimer (μ/mL)
1	53, M	Right cuneus	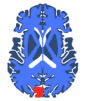	None	SAH, DM, O	-
2	63, F	Right cerebellar hemisphere, pons and thalamus and left inferior parietal lobule	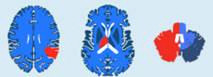	Gliosis	SAH, DM	-
3	65, F	Left superior frontal gyrus and cingulate gyrus	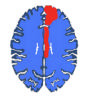	Skull metastasis	HAS, metastatic bladder urothelial carcinoma	4.7 Collected at D1 of symptoms
4	68, M	Bilateral cerebellar hemisphere	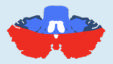	Atheromatosis	SAH, DM, CKD	3.8 Collected at D2 of symptoms
5	72, M	Right cerebellar hemisphere and brainstem	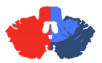	Right vertebral artery dissection	PIS	6.1 Collected at D1 of symptoms
6	72, F	Left inferior frontal gyrus and insula	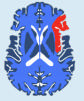	Atheromatosis and gliosis	SAH, DM, O,	-
7	76, M	Right cingulate gyrus	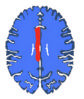	Right occipital lobe gliosis	SAH, S	1.1 Collected at D2 of symptoms
8	76, M	Bilateral posterior territory	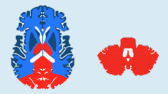	Basilar artery thrombus	None	11.6 Collected at D2 of symptoms
9	79, M	Left cerebellar hemisphere	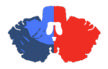	Diffuse gliosis	SAH, AF, prostate cancer	-

F: female; M: male; SAH: systemic arterial hypertension; DM: diabetes mellitus; S: smoking; PIS: previously ischemic stroke; O: obesity; CKD: chronic kidney disease; AF: atrial fibrillation.

All patients were classified as possible COVID-19-associated stroke according to the criteria proposed by Yaghi et al.^5^ with two major and one minor criterion. The d-dimer value was high in all patients in the cohort, with an average value of 5.5 μg/mL and a reference of up to 0.5 μg/mL (Table 1).

## DISCUSSION

Previous studies have shown that acute ischemic stroke (AIS) could be a possible complication in hospitalized patients with COVID-19, while others reported AIS as a viable initial manifestation in those patients^3^ ^,^ ^13^. Our cohort comprised both spectra of patients, some already hospitalized with severe forms of COVID-19 and others who presented at the hospital with acute neurological signs before the pulmonary manifestations, reinforcing the potential link between COVID-19 and cerebrovascular events. The time course of the patients in this cohort reinforces that neurovascular manifestation can begin at any time during the disease, considering the onset of clinical manifestations of neurovascular disease.

As many of the risk factors for the severe form of ­COVID-19 disease are also risk factors for cerebrovascular events, the relationship between these two pathologies can be plausible^8^. Chand et al. showed that in a cohort of 300 severely ill patients, about two-thirds were either obese (grade I) or overweight, 44% were diabetic, 66% had hypertension, and only 19% had no additional risk^14^. Similarly, in our cohort, only one patient had no comorbidity. All others had risk factors for both a severe form of COVID-19 infection and cerebrovascular disease.

Several mechanisms have been proposed to explain a possible association between COVID-19 and AIS. A “cytokine storm”, which refers to an overproduction of inflammatory factors and ultimately induces coagulopathy and vascular endothelial dysfunction, has been found in severe-ill patients^15^. The attributed possible mechanism is either a direct invasion by SARS-CoV-2 or the cytokine storm^15^. A prothrombotic state is installed and can explain (at least in part) the high incidence of ischemic vascular complications, which may paly a synergistic role in patients with comorbidities^15^ ^,^ ^16^. The occurrence of acute cerebrovascular events, especially those of ischemic causes, have been widely reported in the literature, but other etiologies such as inflammatory/infectious, immunological, and even extrapyramidal presentations have also been described^15^. In our sample, we found only cases with ischemic vascular etiology, possibly because our screening of symptomatic neurological patients was performed only with tomographic study due to limited access to more advanced studies, such as magnetic resonance imaging, which reduces the sensitivity in detecting other etiologies.

Vogrig et al.^8^ proposed a revised definition of COVID-19-associated stroke to make this association more objective. It classifies the condition into three degrees of certainty: possible, probable, and confirmed. Although all patients in our study were categorized as possible COVID-19-associated strokes, mainly because they all had risk factors for cardiovascular events, most had a pattern of involvement described as typical for COVID-19, which includes multi-territorial involvement, occlusion of large vessels, and predisposition to posterior circulation^6^ ^,^ ^16^ ^,^ ^17^.

Brazil is one of the countries most affected by the pandemic, but the number of patients with neurovascular syndromes in our study (3%) was not higher than in other countries where the first wave of the disease occurred earlier: China 6%^3^; USA 1.1%^18^; Italy 2.5%^19^; Germany 2%^20^. However, the incidence of 3% may be underestimated due to the predominant use of computed tomography, which has limited sensitivity to subtle lesions.

This study demonstrated the epidemiological aspects of neuroimaging findings in patients with COVID-19 in two tertiary-care Brazilian hospitals. Although the correlation between COVID-19 and stroke is still uncertain, there is a growing number of cases being reported in the literature. Therefore, the authors reinforce the importance of being aware of this situation both in severe hospitalized patients and in patients that present to the emergency room with acute neurological symptoms, even in the elderly.

Nonetheless, it is important to mention that selection biases, which were frequent in the observational analyses, could have had an impact on the results of this study. Furthermore, brain MRI and contrast CT were performed in only a minority of patients due to the availability in the emergency room, which compromised the diagnostic performance.

Further studies are needed to establish a stronger correlation between imaging findings and COVID-19-associated stroke pathophysiology, which will help to develop more effective methods to prevent and treat this complication.
